# Multisystem Proteinopathy Due to *VCP* Mutations: A Review of Clinical Heterogeneity and Genetic Diagnosis

**DOI:** 10.3390/genes13060963

**Published:** 2022-05-27

**Authors:** Gerald Pfeffer, Grace Lee, Carly S. Pontifex, Roberto D. Fanganiello, Allison Peck, Conrad C. Weihl, Virginia Kimonis

**Affiliations:** 1 Hotchkiss Brain Institute, Department of Clinical Neurosciences, Cumming School of Medicine, University of Calgary, Calgary, AB T2N 4N1, Canada; cspontif@ucalgary.ca; 2 Alberta Child Health Research Institute, Department of Medical Genetics, Cumming School of Medicine, University of Calgary, Calgary, AB T2N 4N1, Canada; 3 Division of Genetic and Genomic Medicine, Department of Pediatrics, University of California Irvine Medical Center, Orange, CA 92868, USA; glee1204@gmail.com (G.L.); vkimonis@hs.uci.edu (V.K.); 4 Oral Ecology Research Group, Faculty of Dental Medicine, Université Laval, Quebec City, QC G1V 0A6, Canada; roberto-dalto.fanganiello@greb.ulaval.ca; 5 Cure VCP Disease, Inc., Americus, GA 31709, USA; allison@curevcp.org; 6 Department of Neurology, Washington University School of Medicine, St. Louis, MO 63110, USA; weihlc@wustl.edu

**Keywords:** VCP, multisystem proteinopathy, myopathy, dementia, amyotrophic lateral sclerosis, genetics, genotype-phenotype correlation

## Abstract

In this work, we review clinical features and genetic diagnosis of diseases caused by mutations in the gene encoding valosin-containing protein (VCP/p97), the functionally diverse AAA-ATPase. VCP is crucial to a multitude of cellular functions including protein quality control, stress granule formation and clearance, and genomic integrity functions, among others. Pathogenic mutations in *VCP* cause multisystem proteinopathy (VCP-MSP), an autosomal dominant, adult-onset disorder causing dysfunction in several tissue types. It can result in complex neurodegenerative conditions including inclusion body myopathy, frontotemporal dementia, amyotrophic lateral sclerosis, or combinations of these. There is also an association with other neurodegenerative phenotypes such as Alzheimer-type dementia and Parkinsonism. Non-neurological presentations include Paget disease of bone and may also include cardiac dysfunction. We provide a detailed discussion of genotype-phenotype correlations, recommendations for genetic diagnosis, and genetic counselling implications of VCP-MSP.

## 1. Introduction

Valosin-containing protein (VCP) is encoded by *VCP* on chromosome 9 and it is implicated in numerous human disease phenotypes with autosomal dominant inheritance. VCP is a highly conserved, ubiquitously and abundantly expressed ATPase. Indeed, VCP may make up as much as 1% of all cytoplasmic protein [1]. VCP mediates ubiquitin-dependent cellular processes through the ubiquitin-proteasome system (UPS) [2], protein quality control [3,4], transcription factor processing [5], membrane fusion [6], cell cycle control [7], and regulation of autophagy [8,9].

Over 50 heterozygous missense mutations in *VCP* have been identified in patients with multisystem proteinopathy 1 (MSP1), an autosomal dominant, adult-onset progressive disorder, also known as inclusion body myopathy associated with Paget disease of bone and frontotemporal dementia (IBMPFD), or VCP disease (hereafter referred to as VCP-MSP) [10,11]. The major pathological feature seen in VCP-MSP is the presence of ubiquitin-positive protein aggregates in muscle tissues of patients affected by inclusion body myopathy (IBM). Intranuclear TDP-43+ inclusions are demonstrated in patients with frontotemporal dementia (FTD) and/or amyotrophic lateral sclerosis (ALS) [12]. In Paget disease of bone (PDB), abnormally large osteoclasts containing nuclear inclusions resemble the muscle pathology findings [13]. On the whole, the pathological findings of VCP-MSP suggest that abnormal protein clearance is the major molecular mechanism affected by *VCP* mutations causing human disease. In other forms of MSP (recently reviewed) [11], it has also been suggested that the common molecular mechanism relates to the disruption of two major protein clearance pathways: the UPS and autophagy [14]. However, future studies may identify others among VCP’s diverse molecular mechanisms that are relevant to MSP and other human diseases. The molecular functions of VCP and disease pathogenesis have recently been reviewed [9].

In this review, we provide an overview of the complex clinical presentations of VCP-MSP, with guidance for genetic diagnosis of this complex condition. A special discussion of the complexities surrounding genetic diagnosis and counselling in VCP-MSP is also included.

## 2. Genetic Differential for VCP-MSP

The majority of genetically diagnosed families with MSP are associated with *VCP* mutations [15], although several other genes are associated with MSP phenotypes that may be indistinguishable from VCP-MSP. Mutations in the genes encoding heterogeneous nuclear riboproteins are, respectively, associated with MSP2 and MSP3 [16], although these are thought to be extremely rare [15], with very few reported cases of either disease. MSP2 is caused by mutations in *HNRNPA2B1*, a gene encoding two ribonucleoproteins by alternative splicing (HNRNPA2 and HNRNPB1), which are required for diverse RNA stabilization, splicing, and translation functions, as well as DNA integrity functions [17]. Most of the phenotypes associated with VCP-MSP have been identified in MSP2 patients (myopathy, FTD, ALS, and PDB) [16]. MSP3 is associated with mutations in *HNRNPA1*, a gene encoding a nuclear riboprotein with diverse RNA functions and telomere maintenance [18]. MSP3 presents with ALS, myopathy, and PDB phenotypes [16], including some atypical phenotypes such as flail arm presentation of ALS [19].

MSP4 is caused by mutations in *SQSTM1*, a gene encoding the p62/SQSTM1 protein, a selective autophagy receptor with various other roles in the UPS system and apoptosis [20]. MSP4 has been associated with diverse phenotypes including myopathy [21], PDB [22], ALS [23] and FTD [22]. Interestingly, an uncommon polymorphic variant in *TIA1* appears to modify the phenotype of *SQSTM1* mutations, shifting the penetrance toward myopathy and manifesting as a late-onset, milder phenotype resembling Welander distal myopathy [24,25]. TIA1 is an RNA-binding protein critical to the cellular stress response [26].

Other genes have been associated with phenotypes resembling MSP and should be considered in the differential diagnosis. *MATR3* encodes Matrin 3, a DNA- and RNA-binding protein [27]. *MATR3* mutations are linked to familial myopathy with ALS [28], as well as sporadic ALS disease [29,30]. It was also suggested that some of the familial ALS patients may have had associated dementia, though the clinical presentation was not described [28]; the presence of FTD associated with *MATR3* mutations is at best very rare [31] and there is only a single well-described case in the literature [32]. There are no cases reported of *MATR3* mutations with PDB. *TIA1* is associated with Welander distal myopathy [33], caused by a founder mutation present in Scandinavia [34]. More recently, a mutation in *TIA1* was associated with ALS and FTD [35], but there has been uncertainty with this finding [36,37]. *OPTN* encodes Optineurin, an adaptor protein with numerous protein interactions involved in vesicular trafficking [38]. *OPTN* mutations are another cause of monogenic ALS [39] that appears to have a relationship to other phenotypes resembling MSP, since variation on *OPTN* is a risk factor for sporadic PDB [40] and has also been connected to cases of FTD [41].

## 3. VCP and Genotype-Phenotype Correlations

Mutations in *VCP* cause a spectrum of phenotypes presenting in adulthood, which may coexist with each other in the same patient. We summarize the different clinical features which may appear as manifestations of *VCP* mutations in Table 1. Myopathy is the most common feature and is present in approximately 90% of patients [42]. This most frequently manifests as proximal weakness but variations in the phenotype are described. PDB is present in up to 50% of patients [43] and is caused by altered bone turnover, resulting in bone deformities and pathological fractures. FTD develops in 30% of cases, characterized by early onset and rapidly progressive cognitive syndrome with early behavioral and language deficits [42]. Approximately 10% of patients develop ALS [42], in which there is multifocal degeneration of motor neurons in the brain and spinal cord causing progressive weakness. Other rare and atypical phenotypes have also been described including Charcot-Marie-Tooth disease type 2Y [44,45], hereditary spastic paraplegia [46], and variations on the myopathy phenotype resembling facioscapulohumeral muscular dystrophy, limb-girdle muscular dystrophy, scapulohumeral muscular dystrophy, oculopharyngeal muscular dystrophy, and distal myopathy [12,47]. Other neurodegenerative conditions may be more common in patients with *VCP* mutations, including Parkinson disease and Alzheimer disease [42]. Cardiomyopathy is described as an important feature in case series [48] but the prevalence of cardiac dysfunction is unknown. Expert opinion also cites respiratory muscle weakness as an important consideration for screening and management [49,50]. This broad clinical heterogeneity means that VCP-MSP should be considered in numerous different clinical presentations.

**Figure 1:**
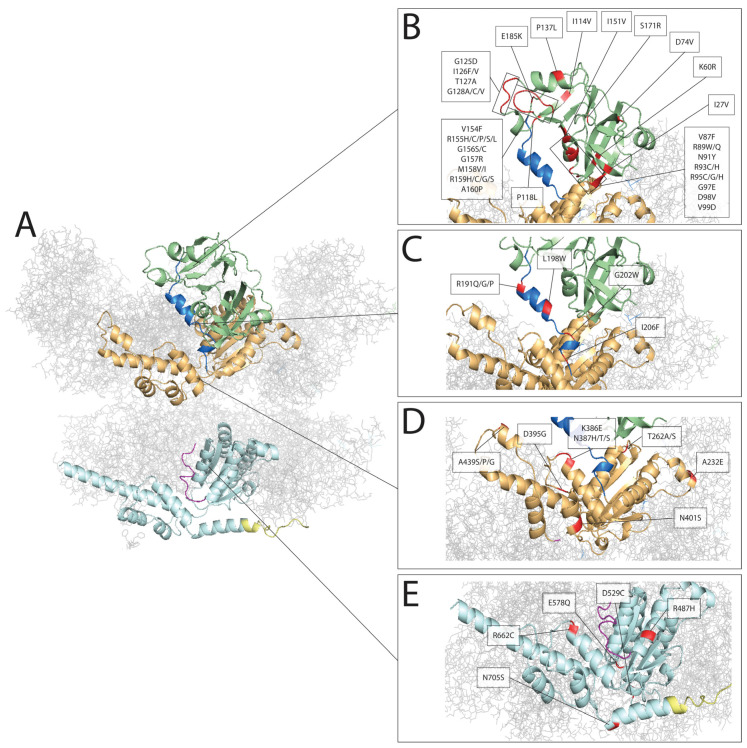
Pathogenic mutations represented on the 3D model of VCP. (**A**) Three-dimensional representation of the VCP hexamer. We have color-coded individual domains from a single VCP monomer for ease of reference in subsequent images: Green for the N-terminal domain, blue for the N-D1 linker, yellow for the D1 domain, purple for the D1-D2 linker, light blue for the D2 domain, yellow for the C-terminal domain, with grey lines representing the other 5 structural oligomers. In red, we have represented the locations of pathogenic variants in VCP, showing the positions in the N-terminal domain (**B**), linker domain (**C**), D1 domain (**D**), and D2 domain (**E**). This figure was generated using open source material available from Swiss model [108] and PyMOL [109] based on the proposed structure of the VCP hexamer [110].

Genotype-phenotype correlation remains a major challenge. There have been major efforts to compile clinical and genetic information from large cohorts of patients, but one of the major limitations is that nearly three-quarters of reported cases are caused by mutations at a single codon for the arginine residue at position 155 [42]. So, although there is much variability for these mutations at position 155, with larger numbers, it may become evident that there is a clear phenotypic correlation for mutations at other residues. Thus far, clear genotype-phenotype correlations appear to be limited. The mutations associated with Charcot-Marie-Tooth disease type 2Y appear to cause restricted phenotypes that differ substantially from classical multisystem involvement of multisystem proteinopathy [44,45,51]. Extended follow-up of these patients may still identify some of the classical VCP-MSP manifestations over time. Two mutations at position 395 also appear to be strongly associated with dementia as a single system disorder [52,53], although again this is based on small case numbers and extended follow-up may identify other associations. We have provided a summary of previously reported pathogenic variants in *VCP* with their genotype-phenotype-mechanistic correlations, organized by their VCP domain in Table 2. These different mutations are also pictorially represented showing their positions in the postulated three-dimensional conformation of the VCP hexamer in Figure 1.

**Table 2:** Mutations in *VCP* organized by domain, and genotype-phenotype correlation.

AA Substitution	Nucleotide Mutation	Domain	Phenotype or Disease	Inheritance	Reference
D6V	c.17A>T	N-Domain Cofactor and Substrate Binding	Myalgia, myopathy, arrhythmia	AD	[54]
I27V	c.79A>G		IBM, PDB, FTD	AD	[55,56]
K60R	c.179A>G		ALS/FTD	SP	[57]
D74V	c.221A>T		Chorea; FTD	AD	[58]
V87F	c.259G>T		IBM	SP	[59]
R89W	c.265C>T		IBM, FTD	SP	[60]
R89Q	c.266G>A		ALS	SP	[61]
N91Y	c.271A>T		IBM, ALS, FTD	AD	[62]
R93C	c.277C>T		IBM, PDB, FTD	AD	[63]
R93H	c.278G>A		HSP	SP	[64]
R95C	c.283C>T		IBM	SP	[65]
R95G	c.283C>G		IBM, PDB, FTD, ALS	AD	[10,42]
R95H	c.284G>A		FTD	UN	[66]
G97E	c.290G>A		IBM, PDB, FTD	AD	[51,67]
D98V	c.293A>T		ALS, FTD	SP	[68]
V99D	c.296T>A		PDB, FTD	AD	[69]
I114V	c.340A>G		IBM (distal arm), ALS	SP	[70,71]
P118L	c.353C>T		FTD	AD	[69]
G125D	c.374G>A		IBM, PDB, FTD	AD	[69,72]
I126F	c.376A>T		IBM	AD	[73]
I126V	c.376A>G		IBM, FTD	SP	[59]
T127A	c.379A>G		FTD	SP	[74]
G128A	c.383G>C		IBM, PDB, FTD	AD	[42,75]
G128C	c.382G>T		IBM	SP	[75]
G128V	c.383G>T		IBM, PDB, FTD	AD	[76]
P137L	c.410C>T		IBM (distal), PDB, FTD	AD	[77]
I151V	c.451A>G		ALS	SP	[78]
V154F	c.460G>T		FTD	AD	[79]
R155H	c.464G>A		IBM, PDB, FTD, ALS	AD	[10]
R155C	c.463C>T		IBM, PDB, FTD, ALS, PD	AD	[10,80]
R155P	c.464G>C		IBM, PDB, FTD	AD	[10]
R155S	c.463C>A		IBM, PDB, FTD	AD	[81]
R155L	c.464G>T		IBM, PDB, FTD, ALS, SNH	AD	[82]
G156S	c.466G>A		IBM, PDB, FTD	AD	[83,84]
G156C	c.466G>T		ALS	AD	[85]
G157R	c.469G>C		IBM, PDB, FTD, SNH	AD	[86]
M158V	c.472A>G		PDB, ALS, FTD	AD	[87,88]
M158I	c.474G>A		IBM, PDB, ALS	AD	[42,75]
R159H	c.476G>A		IBM, PDB, FTD, ALS	AD AR	[70,72,89,90]
R159C	c.475C>T		IBM, PDB, FTD, ALS, Parkinson disease, HSP	AD	[80,91,92,93]
R159G	c.475C>G		FTD, ALS	AD	[94]
R159S	c.475C>A		FTD	AD	[88]
A160P	c.478G>C		IBM, PDB, FTD, ALS	AD	[42,75]
E185K	c.553G>A		CMT2		[44]
R191Q	c.572G>A	N-D1 Linker	IBM, PDB, FTD, ALS	AD	[10]
R191G	c.571C>G		IBM, ALS	AD	[80]
R191P	c.572G>C		ALS	AD	[95]
L198W	c.593T>G		IBM, PDB, FTD	AD	[96]
G202W	c.604G>T		IBM, FTD	AD	[97]
I206F	c.616A>T		IBM, PDB, FTD	AD	[98]
A232E	c.695C>A	D1 Oligomerization Domain Heat Enhanced ATPase Domain	IBM, PDB, ALS	AD	[10]
T262A	c.784A>G		IBM, PDB, FTD, Parkinsonism	AD	[99]
T262S	c.785C>G		FTD	AD	[88]
K386E	c.1156A>G		Myopathy	NR	[100]
N387H	c.1159A>C		IBM, FTD	AD	[96]
N387T	c.1160A>C		ALS	SP	[101]
N387S	c.1160G>A		IBM, PDB, FTD	AD	[102]
D395G	c.1184A>G		FTD	AD	[53,79]
D395A	c.1184A>C		FTD	AD	[52]
N401S	c.1202A>G		Alzheimer Dementia	SP	[74]
A439S	c.1315G>T		IBM, PDB	AD	[81]
A439P	c.1315G>C		IBM, PDB, FTD	AD	[103]
A439G	c.1316C>G		IBM, FTD	AD	[97]
R487H	c.1460G>A	D2 Major ATPase Domain	FTD, ALS	AD	[104]
E578Q			IBM	UN	[105]
D592N	c.1774G>A		ALS	AD	[94]
R662C	c.1984C>T		ALS	SP	[101]
N750S	c.2249A>G		ALS	SP	[106]
9:35060456 del (5) Proposed fs at AA 515			Autism	DN	[107]

**Abbreviations:** Amino acid sequence (AA), Autosomal dominant (AD), amyotrophic lateral sclerosis (ALS), Autosomal recessive (AR), Charcot-Marie-Tooth disease type 2 (CMT2), de novo (DN), frontotemporal dementia (FTD), inclusion body myopathy (IBM), Paget disease of bone (PDB), sensorineural hearing loss (SNH), Sporadic (SP), Unknown (UN).

Reasons for the generally poor genotype-phenotype correlation could be related to various factors. For any late-onset genetic disorder, the interaction of environmental factors is certainly possible, and there is precedent from other late-onset neuromuscular conditions [111,112,113]. Host-microbiome interactions may be another way in which the onset and severity of disease can be modified, as is seen from early work in other neurodegenerative conditions [114]. However, it also bears mention that much of the variability may still be genetic: there is some evidence that modifier effects can alter the phenotype, such as APOε4 alleles associated with the FTD phenotype [115]. A recent publication also suggests a possible interaction with an intermediate-length repeat in *ATXN2* and a complex neurodegenerative phenotype [116]. Future studies may also consider other possible modifiers that have been identified from studies of animal models or related disorders [24,25,117].

## 4. Genetic Diagnosis of VCP-MSP

The diagnosis of VCP-MSP is confirmed by the identification of a pathogenic mutation in *VCP* using genetic testing. Diagnosis of VCP-MSP should be considered in patients having one or more of the clinical presentations described in Table 1. In the presence of an autosomal dominant family history, the likelihood of diagnosing a genetic disorder is much greater. However, cases without family history may still be caused by *VCP* mutations due to variations in disease penetrance and expressivity, attribution of alternate diagnoses, or presence of de novo mutations. When interviewing patients for family history, it is important to consider the above-mentioned clinical variability and ask about muscle weakness, bone disease, dementia and ALS, since phenotypes may vary and do not correlate with genotype. As mentioned above, the exception is Charcot-Marie-Tooth disease type 2Y, which appears to be correlated to particular *VCP* mutations [44,45,51]. Parkinson disease and Alzheimer disease have been associated with *VCP* mutations, although it is important to be aware that these conditions are also common in the aged population. Some specific phenotypes may have higher likelihood for a diagnosis with VCP-MSP, for example, ALS is a relatively common disease compared with the prevalence of VCP-MSP; therefore, the likelihood of identifying a *VCP* mutation in a sporadic case of ALS is low. Myopathy with onset in adulthood is extensively heterogeneous with a low likelihood of obtaining any genetic diagnosis; therefore, isolated myopathy on its own would not suggest a high likelihood of identifying VCP-MSP. However, myopathy is the most common presentation of VCP-MSP and it is possible that some cases of VCP-MSP are misdiagnosed as sporadic inclusion body myositis based on reactivity to NT5C1A antibodies [118]. Early-onset FTD is very rare and may portend a higher likelihood of identifying a causative gene mutation.

### 4.1. Genetic Testing Methods

There are several testing strategies generally available to clinicians, which broadly speaking can be divided into three categories: (a) targeted single gene testing, (b) multi-gene panel sequencing, and (c) unbiased sequencing (exome or whole genome).

*Single gene/variant testing*: This is when only *VCP* is sequenced, or if specific variants in *VCP* are sequenced as a targeted investigation. Generally, this type of approach is only used for testing of family members in which a diagnosis of VCP-MSP is already established in the family. We do not suggest single gene testing in situations where there is not a known diagnosis of VCP-MSP in the family; this is due to heterogeneity of clinical syndromes associated with VCP-MSP that includes a vast genetic differential diagnosis. Therefore, it is more cost- and time-effective to use parallel sequencing approaches for diagnosis of undifferentiated cases. This also applies to cases in which a diagnosis of VCP-MSP is highly likely (e.g., dominant history with PDB, FTD, and myopathy) because there remains a differential diagnosis with other multisystem proteinopathy genes (as per Section 2 above).

*Panel sequencing*: This is when multiple genes are tested simultaneously, usually applying short-read parallel sequencing methods. Panels are offered by numerous genetic testing companies with clinical certification. Since *VCP* mutations are associated with so many phenotypes, it is included in panels to investigate genetic causes of myopathy, dementia, and ALS. However, the clinician should be aware that *VCP* may not be included in panels from all companies. Some panels for skeletal abnormalities, spastic paraplegia, and neuropathies do not routinely include *VCP*. Since the content of sequencing panels is adjusted over time, panels will hopefully be more inclusive in the future; thus, in patients without an established genetic diagnosis, re-evaluating that *VCP* was indeed on the initial panel may be warranted, leading to repeat panel testing. In general, for undifferentiated cases, panel sequencing will usually be the most readily available test and the most suitable investigation for diagnosing patients with VCP-MSP or other genetic conditions in the differential diagnosis.

*Unbiased sequencing*: Genomic approaches that sequence all coding regions (exome sequencing) or the entire genome are typically reserved for use by medical genetic specialists or in research settings. As long as the read depth and coverage are sufficient, *VCP* will always be sequenced in cases receiving this type of testing. However, because all other genes are also sequenced, this can result in challenges in the interpretation if multiple potentially relevant variants are identified. This also has a high likelihood of identifying incidental genetic findings that have special genetic counselling implications. There is evidence indicating a higher diagnostic yield for unbiased sequencing approaches compared with panel sequencing for genetic diseases, and this may also be the case for VCP-related disorders which can produce unexpected phenotypes.

Our recommendation is that in most situations, multi-gene panel sequencing will be the best option for testing of undifferentiated cases, whereas single gene testing should be reserved for cases with a known family history of VCP-MSP. Broad genetic testing (such as panel or exome/genome) also has the advantage of being able to identify dual (or multiple) genetic diagnoses, which are believed to occur in ~5% of patients with Mendelian genetic disorders [119]. We may eventually become aware of non-Mendelian gene modifier effects or interactions [120], for which broader sequencing approaches will also be necessary.

We recommend that *VCP* should be included in genetic sequencing panels for a broad range of phenotypes, including myopathy, skeletal disorders, dementia, ALS, spastic paraplegia, and neuropathy, to aid in the identification of a definitive diagnosis for as many patients as possible.

### 4.2. Access to Genetic Testing

Genetic testing is increasingly accessible and convenient. Many test providers give the option of providing a blood sample or a sample collected at home (saliva or buccal DNA kits). Coverage for the costs of genetic testing differs widely across jurisdictions; however, many companies will offer a reduced price for “patient pay” testing, when insurers or health systems do not cover costs. There are also sponsored genetic testing programs that offer testing free of charge, with several that are currently active for testing of patients with myopathy and neuropathy.

### 4.3. Complementary Testing and Reverse Clinical Correlation

Our recommendation is to use genetic testing as the means to reach definitive diagnosis. Additional tests such as muscle biopsy, creatine kinase enzyme levels, neurophysiology (nerve conduction testing and electromyography), and skeletal surveys can give supportive information for the diagnosis of VCP-MSP. However, none of these investigations are specific to *VCP* mutations and, in many cases, typical abnormalities may not be present even in genetically confirmed cases of VCP-MSP [121].

Muscle biopsy findings associated with VCP-MSP classically show rimmed vacuoles, with ubiquitin positive and tubulofilamentous inclusions on electron microscopy [121]. However, these characteristic findings were seen in only 39% of cases from the largest series of muscle biopsies in VCP-MSP [12]. Nonspecific myopathic changes are frequently identified.

MRI of the muscles is increasingly used to differentiate underlying causes of myopathy and aid in genetic differential diagnosis [122]. There are a few previously reported cases with muscle MRI findings in VCP-MSP [122,123,124] and further study will help to better define the range of findings as well as determining their sensitivity and specificity.

These tests may be carried out to investigate patient symptoms, but increasingly these may also be performed as screening investigations or as “reverse clinical correlation” in patients who have a *VCP* mutation identified in genetic testing. The former situation is familiar and straightforward, where an undifferentiated patient with myopathy symptoms and signs on examination is referred for a muscle biopsy, or a patient with muscle atrophy and fasciculations is referred for electromyography. When additional testing is requested in a patient already diagnosed with genetic testing, this is often variably based on clinician experience and individual judgement. For example, a patient with myopathy who is found to have a pathogenic *VCP* mutation on a multi-gene panel may be referred for electromyography, skeletal survey, and MRI of the head, to identify other features in the clinical spectrum of VCP-MSP prior to the development of symptoms. This can have particular value for early identification of PDB, which is responsive to therapy with bisphosphonates [125]. Identification of other features such as ALS may have value for prognostication, or as a baseline investigation that can be a useful reference in case of later clinical deterioration.

### 4.4. Interpreting Genetic Test Results

The general principles of interpreting genetic variants in *VCP* are similar to those for other diseases. Most commonly, the American College of Medical Genetics (ACMG) criteria [126] are applied to reach a determination.

**(1)**  Positive: A positive result means that a variant in *VCP* known to be associated with VCP-MSP has been identified in the patient (i.e., a “pathogenic” or “likely pathogenic” variant). Thus far, all confirmed pathogenic variants in *VCP* are missense. A positive result may confirm the diagnosis in a person who meets clinical criteria or provide a pre-symptomatic diagnosis for an unaffected individual. If an unaffected individual has a positive result, there is a 90% chance that he or she will experience one or more of the features of VCP-MSP by the age of 45 [121]. Onset of symptoms is age-dependent and pre-symptomatic patients should be monitored by a healthcare professional on a regular basis.
**(2)**  Negative: A negative result means that no pathogenic variant has been identified in *VCP*. Depending on the test that was performed, and the purpose of testing, the implications of this result may vary:
  **(a)**  Affected Individual: This means their condition is not caused by a mutation in *VCP*, but it remains possible that a mutation in another gene that was not tested could be responsible.
  **(b)**  Unaffected individual in a family with a known pathogenic *VCP* mutation: In this situation, a negative result means that the person does not have the mutation and, therefore, will not develop VCP-MSP. The individual will not require follow-up care or monitoring for symptoms as they are confirmed to be unaffected and cannot pass it on to their children.
  **(c)**  Affected individual in a family having a pathogenic *VCP* mutation: Individuals who are symptomatic but are non-carriers of the familial mutation may also have a “phenocopy” syndrome that should be considered in this situation [127]. Causes of phenocopy syndromes could be due to diagnostic confusion with acquired disease, a different genetic diagnosis, or non-organic symptoms.

### 4.5. Variant of Unknown Significance

A variant of unknown significance (VUS) is a change in *VCP* that has certain characteristics of a pathogenic mutation (e.g., not present in controls and alters the amino acid sequence) but has not previously been associated with human disease. This result means that insufficient data exist to support whether the variant is benign or pathogenic. If it has been identified previously, the variant may have conflicting reports of pathogenicity. The lab evaluates the variant to determine the consequence of the amino acid sequence, level of conservation and frequency in population databases, and will provide this information in the report. Because of the uncertainty of their relationship to disease, VUS are not clinically actionable. VUS can sometimes be reclassified based on results from familial segregation (when sufficient numbers of affected/unaffected individuals are available) or research-based investigations. The lab may also reclassify a VUS after new information becomes available; many labs will issue updated reports when this occurs, but it can also be appropriate to recontact the lab time for VUS reanalysis. In general, genetic testing reports will attempt to integrate all available current information to determine a variant’s classification, but it is worth mentioning that information on public databases such as ClinVar or HGMD may not necessarily be up to date. The ACMG criteria also contain additional discussion of VUS for further reference [126].

## 5. Genetic Counselling

For undifferentiated cases, ordering physicians should ensure patients are aware that genetic test results may have implications for other family members and that depending on results, formal genetic counselling may be recommended for themselves as well as other family members. In these respects (and relating to other issues discussed below), genetic testing has been considered to have broader implications than other investigations (“genetic exceptionalism”), although in the context of wide availability of unbiased or parallel sequencing tests, this topic has also been subject to evolving debate in the literature [128,129]. Given the high likelihood of identifying VUS with panel or exome/genome sequencing, patients should also be prepared for the possibility of an indeterminate result.

Access to formal genetic counselling in some regions has not grown at the same rate as access to genetic testing [130]. Various approaches have been recommended including the use of telehealth to improve access to these resources [131]. There are also online resources available that can provide genetic counselling, and evidence shows this approach can be highly satisfactory for patients [132].

### 5.1. Risk to Other Family Members

Each cell in the body has two copies of the *VCP* gene. VCP-MSP is an autosomal dominant disorder, meaning that one pathogenic mutation in either copy of *VCP* is sufficient to cause the disease. If a parent has a pathogenic *VCP* variant, then each of his or her children will have a 50% probability (1 in 2 chance) of inheriting the variant, regardless of sex. If a patient has a pathogenic *VCP* variant, each full sibling has a 50% probability (1 in 2 chance) of carrying the variant as well, unless it is a confirmed de novo variant.

The known *VCP* pathogenic variants are highly penetrant, with approximately 90% of mutation carriers developing symptoms by the age of 45 [121]. Variability in severity and age of onset is seen, even among members of the same family with the same variant. For example, the disease may progress at different rates, and family members may have variable manifestations such as PDB, FTD, and/or ALS [62]. This variability is not currently understood but may be related to genetic modifiers or environmental factors.

### 5.2. Pre-Symptomatic Testing

If there is a known *VCP* variant in a family with VCP-MSP, testing is available for pre-symptomatic individuals. Individuals may wish to pursue pre-symptomatic testing to resolve uncertainty regarding their risk of the disease, including reproductive, career, or other lifestyle planning [133]. In general, such testing should be a carefully considered decision following formal pre-test genetic counselling and thorough review of the implications of a positive or negative result [134]. Post-test genetic counselling is equally important for individuals who test positive or negative with pre-symptomatic testing [135]. Offering mental health supports can be highly beneficial, again irrespective of the test result [135].

One study specifically considered pre-symptomatic testing for individuals from families with known VCP-MSP. Anxiety levels were initially high before and after testing, but back to their normal anxiety level one year following testing [136]. Predictive testing for pre-symptomatic individuals is an emotionally complex process for the patient, and testing protocols for other conditions such as Huntington disease may be referenced and adapted for VCP-MSP [137]. An unaffected individual that tests negative while other family members test positive may feel guilt for their unaffected status.

The presence of a pathogenic *VCP* variant in an asymptomatic individual identifies them as a pre-symptomatic patient. Because of the above-mentioned high penetrance for *VCP* mutations, pre-symptomatic individuals should be monitored for the development of disease manifestations.

The diagnosis of VCP-MSP poses some unique challenges compared to other genetic disorders. The most common clinical presentation will be myopathy, which is generally slowly progressive. In the course of discussing a VCP-MSP diagnosis, it is also necessary to mention the chance of eventually developing a more severe disorder such as frontotemporal dementia or ALS. This effectively results in a situation where a symptomatic patient with VCP-MSP also simultaneously becomes a pre-symptomatic patient for a separate, severe, variable-penetrance disease. There may be additional unexpected psychosocial consequences to patients in this situation, for example, the myopathy patient who repeatedly becomes concerned that they have started developing ALS if they notice any hand or bulbar symptoms at all or likewise are repeatedly concerned that they have started to develop FTD if they perceive anything that could be a cognitive symptom. We emphasize the value of continued follow-up with patients and proactive engagement of mental health resources.

### 5.3. Genetic Discrimination

Fear of genetic discrimination and protection of personal data are major factors present among individuals considering genetic testing [138]. Furthermore, a person’s culture, race, and ethnicity may influence their willingness to pursue genetic testing, so it may be important to address genetic discrimination fears and cultural influences during genetic counselling [139].

Genetic discrimination and the laws to protect patients vary widely depending on a patient’s country, and many countries have started to adopt laws to protect patients [140]. Canada’s Genetic Non-Discrimination Act (GNDA) prohibits companies and employers from requiring genetic testing or the results of genetic tests. In Europe, a collection of guidelines have been adopted concerning the use of genetic information for insurance purposes [141]. In the United States, all individuals pursuing genetic testing are protected against genetic discrimination by the Genetic Information Nondiscrimination Act of 2008 (GINA) [142]. This act protects individuals from discrimination based on their genetic status with employment and health insurance. Employers are prevented from using employee genetic information to make employment decisions and are prevented from requesting genetic information from employees. Health insurers are prevented from denying coverage, making decisions on premiums or coverage based on genetic information, or requesting genetic information from individuals or family members. GINA does not include protections from life, disability, or long-term care insurance discrimination. There are exceptions to the employment and health insurance protections explained above, and relevant details should be discussed with patients depending on their specific circumstances and jurisdiction.

## 6. Conclusions

The complexity of VCP’s diverse molecular functions is also mirrored by the variability in clinical dysfunction caused by pathogenic variants in *VCP*. The relationship between specific molecular functions of VCP and the spectrum of clinical presentations remains poorly understood and, in general, genotype–phenotype correlation is still difficult to demonstrate. Because of this, the diagnosis of VCP-MSP is best achieved by the inclusion of *VCP* in numerous sequencing panels including myopathy, PDB, FTD, ALS, axonal Charcot-Marie-Tooth disease, and/or spastic paraplegia. The inclusion of *VCP* in panels for common neurodegenerative disorders such as Alzheimer and Parkinson disease may also be reasonable, but clinical judgement is required in test result interpretation for these disorders which are common in the aged population. As a late-onset disorder with variable disease severity and no disease-modifying therapy, the implications of genetic testing for patients and family members are complex, and patients benefit from access to genetic counselling to discuss the unique aspects of their test implications.

## Figures and Tables

**Table 1:** Neurological localizations and clinical phenotypes associated with VCP-MSP.

Localization	Phenotype or Disease	Frequency in VCP-MSP
Central nervous system	**Frontotemporal dementia**	30%
	Alzheimer disease	2%
	Parkinson disease	4%
	Spastic paraplegia	Case reports
Peripheral nervous system	**Inclusion body myopathy**	90%
	Amyotrophic lateral sclerosis	10%
	Charcot-Marie-Tooth disease	Case reports
	Sensory polyneuropathy	Case reports
Non-neurological	**Paget disease of bone**	40%
	Cardiomyopathy	Uncertain

## Data Availability

All relevant data have been cited within the document.
